# Interactive Roles for AMPK and Glycogen from Cellular Energy Sensing to Exercise Metabolism

**DOI:** 10.3390/ijms19113344

**Published:** 2018-10-26

**Authors:** Natalie R. Janzen, Jamie Whitfield, Nolan J. Hoffman

**Affiliations:** Exercise and Nutrition Research Program, Mary MacKillop Institute for Health Research, Australian Catholic University, Level 5, 215 Spring Street, Melbourne, Victoria 3000, Australia; natalie.janzen@acu.edu.au (N.R.J.); jamie.whitfield@acu.edu.au (J.W.)

**Keywords:** AMP-activated protein kinase, glycogen, exercise, metabolism, cellular energy sensing, energy utilization, liver, skeletal muscle, metabolic disease, glycogen storage disease

## Abstract

The AMP-activated protein kinase (AMPK) is a heterotrimeric complex with central roles in cellular energy sensing and the regulation of metabolism and exercise adaptations. AMPK regulatory β subunits contain a conserved carbohydrate-binding module (CBM) that binds glycogen, the major tissue storage form of glucose. Research over the past two decades has revealed that the regulation of AMPK is impacted by glycogen availability, and glycogen storage dynamics are concurrently regulated by AMPK activity. This growing body of research has uncovered new evidence of physical and functional interactive roles for AMPK and glycogen ranging from cellular energy sensing to the regulation of whole-body metabolism and exercise-induced adaptations. In this review, we discuss recent advancements in the understanding of molecular, cellular, and physiological processes impacted by AMPK-glycogen interactions. In addition, we appraise how novel research technologies and experimental models will continue to expand the repertoire of biological processes known to be regulated by AMPK and glycogen. These multidisciplinary research advances will aid the discovery of novel pathways and regulatory mechanisms that are central to the AMPK signaling network, beneficial effects of exercise and maintenance of metabolic homeostasis in health and disease.

## 1. Introduction

The AMP-activated protein kinase (AMPK) is a heterotrimer composed of a catalytic α subunit and regulatory β and γ subunits, which becomes activated in response to a decrease in cellular energy status. Activation of AMPK results in metabolic adaptations such as increases in glucose uptake and glycolytic flux and fatty acid (FA) oxidation. AMPK activation simultaneously inhibits anabolic processes including protein and FA synthesis. AMPK can also translocate to the nucleus where it regulates transcription factors to increase energy production, meet cellular energy demands and inhibit cell growth and proliferation. Conversely, when energy levels are replete, AMPK activity returns to basal levels, allowing anabolic processes to resume. Given its central roles in cellular metabolic and growth signaling pathways, AMPK remains an appealing target for treating a range of pathologies associated with obesity and aging, including metabolic diseases such as obesity and type 2 diabetes (T2D).

In response to changes in energy supply and demand, glycogen, predominately stored in the liver and skeletal muscle, serves as an important source of energy to maintain metabolic homeostasis. Glycogen is synthesized by the linking of glucose monomers during periods of nutrient excess. In response to energy stress and decreased arterial glucose concentration, rising glucagon levels induce increased hepatic glucose output by promoting the breakdown of glycogen and the conversion of non-glucose substrates into glucose. The newly formed glucose is released into the bloodstream to help restore blood glucose levels. Skeletal muscle glycogen serves as an accessible source of glucose to form adenosine triphosphate (ATP) and to reduce equivalents via glycolytic and oxidative phosphorylation pathways during muscle contraction.

A significant body of evidence demonstrates that AMPK binds glycogen. This physical interaction is mediated by the carbohydrate-binding module (CBM) located within the AMPK β subunit and is thought to allow AMPK to function as a sensor of stored cellular energy. While glycogen is stored in multiple tissues throughout the body, this review will primarily focus on the physical interactions underlying the AMPK β subunit binding to glycogen and its potential functional links to glycogen storage dynamics in the liver and skeletal muscles, as these tissues are central to metabolic and exercise-regulated biological processes. In addition, as the majority of research on this topic has been undertaken in human and mouse model systems, studies in these species will be highlighted. Following a brief background on the regulation of AMPK and glycogen, this review will critically assess the recent advances and focus primarily on studies within the past two decades that have added to our understanding of the physical basis of AMPK-glycogen binding and its potential functional interactions in exercise and metabolism. Key remaining biological questions related to the interactive roles of AMPK and glycogen will be posed along with a discussion of research advancements that are feasible in the next decade with new technologies and experimental models to determine how AMPK-glycogen binding may be therapeutically targeted in health and disease.

## 2. Roles for AMPK and Glycogen in Metabolism

### 2.1. AMPK Activation and Signaling

**Figure 1:**
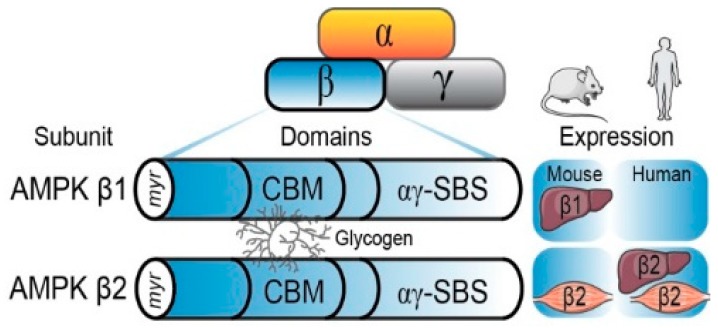
The AMPK is a heterotrimeric protein, consisting of a catalytic α subunit and regulatory β and γ subunits. The β subunit (β1 and β2 isoforms) possesses a glycogen-binding domain (CBM) that mediates AMPK’s interaction with glycogen, an N-terminal myristoylation site (myr) and an αγ subunit binding sequence (αγ-SBS) involved in the heterotrimeric complex formation. Tissue expression of the β1 and β2 isoforms varies between humans and mice, as the β2 isoform is predominately expressed in both human liver and skeletal muscles, while mice predominately express the β1 isoform in the liver and the β2 isoform in the skeletal muscles.

Structural biology-based studies over the past decade have provided new insights into the molecular mechanisms by which AMPK activation is regulated by nucleotides, including changes in AMP:ATP and ADP:ATP ratios (i.e., adenylate energy charge) that occur in response to cellular energy stress [1]. The binding of AMP and ADP to the cystathionine-β-synthase (CBS) domains of the γ subunit promotes AMPK activation through several complementary mechanisms. The binding of AMP promotes AMPK association with liver kinase B1 (LKB1) and the scaffolding protein axin which enhances the effect of T172 phosphorylation [2,3], the primary phosphorylation and activation site in the AMPK α subunit, while simultaneously preventing its dephosphorylation by protein phosphatases [4,5,6,7]. This activation is mediated by myristoylation of the G2 site on the N-terminus of the β subunit (Figure 1), which promotes AMPK association with cellular membranes and LKB1 [2]. Furthermore, the binding of AMP, but not ADP, can cause the allosteric activation of AMPK without T172 phosphorylation [5,7,8,9]. The combination of allosteric activation by nucleotides and increased T172 phosphorylation by LKB1 can increase AMPK activity 1000-fold [10]. Additionally, the phosphorylation of T172 can be regulated by changes in intracellular Ca^2+^ concentrations via the upstream kinase calcium/calmodulin-dependent protein kinase kinase β (CAMKK2) in the absence of changes in adenylate energy charge [11,12,13].

Once activated, AMPK serves as a metabolic ‘switch’ to promote catabolic pathways and inhibit anabolic processes. For example, AMPK increases glucose uptake into skeletal muscle by phosphorylating and inhibiting Tre-2, BUB2, CDC16, 1 domain family, members 1 (TBC1D1) and 4 (TBC1D4), promoting glucose transporter 4 (GLUT4) vesicle translocation to the sarcolemmal membrane [14,15,16,17,18]. AMPK also functions in the regulation of lipids, acutely promoting lipid oxidation and inhibiting FA synthesis, primarily through phosphorylation and the inhibition of acetyl-CoA carboxylase (ACC) [19,20]. At the transcriptional level, AMPK phosphorylates and inhibits sterol regulatory element-binding protein 1, a transcription factor that regulates lipid synthesis [21]. Mitochondrial biogenesis is stimulated by AMPK activity through an increase in the peroxisome proliferator-activated receptor gamma coactivator 1-alpha (PGC-1α) transcription, thereby promoting oxidative metabolism [22]. AMPK can also inhibit anabolic pathways by phosphorylating the regulatory associated protein of the mechanistic target of rapamycin (mTOR) (Raptor) and the tuberous sclerosis complex 2 (TSC2), which in turn inactivates mTOR and prevents the phosphorylation of its substrates [23,24,25].

### 2.2. The AMPK β Subunit and Carbohydrate-Binding Module

The AMPK β subunit exists in two isoforms (β1 and β2) and serves as a scaffolding subunit that binds to the AMPK catalytic α and regulatory γ subunits, playing an important role in the physical stability of the heterotrimer (Figure 1) [9,26,27]. In human and mouse skeletal muscles, the β2 isoform is predominantly expressed (Table 1) [28]. In contrast, the liver β subunit isoform expression differs across the mammalian species: the β1 isoform is predominantly expressed in mice, while β2 is predominantly expressed in humans [29,30]. However, despite isoform differences between species, both the β subunit isoforms contain the CBM which mediates physical AMPK-glycogen interaction and binding. Furthermore, the CBM is highly conserved between species, suggesting that the region possesses evolutionary significance and plays similar roles across species [31,32]. The CBM spans residues 68–163 of the β1 subunit and residues 67–163 of the β2 subunit [32,33] and is nearly identical in structure and sequence in both isoforms, with the major difference being the insertion of a threonine at residue 101 in the β2 CBM [34,35]. This insertion is believed to have occurred early in evolutionary history and provides the β2 CBM with a higher affinity for glycogen [33,34]. However, the reason for this divergence in β subunit isoforms is unknown.

### 2.3. Glycogen Dynamics

A number of proteins are associated with glycogen particles and function as regulators of glycogen synthesis, breakdown, particle size, and degree of branching. Glycogenin initiates glycogen formation and functions as the central protein of the glycogen particle [37]. Glycogen synthase (GS) is the rate-limiting enzyme in glycogen synthesis responsible for attaching UDP-glucose donors together in α-1,4 linkages, the linear links of the glycogen particle. As glucose-6-phosphate (G6P) is a precursor to UDP-glucose, its accumulation is a potent activator of GS, capable of overriding the inhibitory effects of phosphorylation mediated by proteins such as AMPK, glycogen synthase kinase 3, and protein kinase A [38,39]. As its name implies, glycogen branching enzyme (GBE) is responsible for introducing α-1,6 branch points to the growing glycogen particle. The rate-limiting enzyme of glycogen breakdown is glycogen phosphorylase (GP), which is known to be activated by elevated intracellular Ca^2+^, epinephrine and cAMP concentrations [40,41]. When activated, GP degrades the α-1,4 links of glycogen particles and removes glycosyl units from the non-reducing ends of the glycogen particle [42]. The glycogen debranching enzyme (GDE) assists with the degradation of glycogen and is responsible for breaking the α-1,6 links to allow continued GP activity. Without GDE, GP can only degrade the outer tiers of glycogen particles and stops four glucose residues short of the α-1,6 branch point [39]. Further details regarding glycogen synthesis and breakdown are beyond the scope of this review, and readers are referred to other reviews covering this topic [39,43].

### 2.4. Glycogen Localization

Recently, there has been an increasing interest regarding the significance of glycogen’s subcellular localization in skeletal muscles [44,45,46,47,48]. Glycogen can be concentrated beneath the sarcolemma (subsarcolemmal; SS), between the myofibers along the I band near the mitochondria and sarcoplasmic reticulum (intermyofibrillar; interMF), or within myofibers near the triad junction (intramyofibrillar; intraMF) [44,46]. Depletion of these different glycogen pools impacts muscle function and fatigue, such as impairing Ca^2+^ release and reuptake. Therefore, it has been hypothesized that these different pools of glycogen play a significant role in muscle contraction and fatigue beyond their role as an energy substrate [45,47,48].

Human skeletal muscles contain large stores of glycogen, which can exceed 100 mmol glucosyl units/kg wet weight (~500 mmol/kg dry weight) in the vastus lateralis muscle [49,50] and are primarily concentrated in the interMF space [49]. Conversely, rodents tend to have higher stores of glycogen in the liver compared to the skeletal muscle. For example, mice store about 120 μmol/g wet weight in liver and 15–20 μmol glucose/g wet weight in the type IIA flexor digitorum brevis muscle, with the highest concentrations in the intraMF pool [51]. Interestingly, while the relative contributions of intraMF glycogen to total glycogen are different between humans and mice, the intraMF content as a percentage of the total fiber volume is very similar between species [49,51]. Additionally, substrate utilization is different between species during exercise, as humans rely predominately on intramuscular stores and rodents rely on blood-borne substrates [52,53,54,55]. These differences in glycogen storage and utilization between humans and rodents are important considerations in the study design across species when assessing glycogen depletion and/or repletion.

## 3. Molecular Evidence of AMPK-Glycogen Binding

In 2003, it was first demonstrated that recombinant AMPK β1 CBM bound glycogen using a cell-free assay system [31]. Structural prediction and mutagenesis experiments targeting conserved residues within the CBM thought to mediate glycogen binding demonstrated that W100G and K126Q mutations abolished glycogen binding to the isolated β1 CBM, while W133L, S108E, and G147R mutations partially disrupted glycogen binding. Additionally, the AMPK heterotrimeric complex was found to bind glycogen more tightly than the β1 subunit in isolation; however, the reasons for this differential binding affinity remain unclear [31]. In support of these findings, cell-free assays have also revealed that glycogen has an inhibitory effect on AMPK activity [56]. Furthermore, mutation of critical residues in the β1 CBM (W100G, W133A, K126A, L148A, and T148A) ablated glycogen’s inhibition. In these cell-free assays, glycogen with higher branch points had a greater inhibitory effect on AMPK, indicating that glycogen particle size has the capacity to influence AMPK-glycogen interactions [56]. It was also observed that glycogen particles co-localized with the β subunit of AMPK in the cytoplasm of CCL13 cells [57]. A follow-up structural-based study determined the CBM crystal structure in the presence of β-cyclodextrin and confirmed that AMPK indeed interacts with glycogen [32]. Additional experimental approaches such as immunogold cytochemistry have also shown that the AMPK α and β subunits of rat liver tissue are associated with the surface of glycogen particles in situ, providing further molecular evidence supporting this concept of physical AMPK-glycogen interaction [58].

In addition to its role in binding glycogen, the CBM of the β subunit also physically interacts with the kinase domain of the catalytic α subunit, forming a pocket, referred to as the allosteric drug and metabolite (ADaM) site. Small molecule AMPK activators such as A-769662, a β1 subunit specific activator, bind to this site and directly activate AMPK [27,59]. However, to date, any connection of A-769662’s subunit specificity in relation to glycogen has been highly speculative and further research is required to establish potential direct links. The ADaM site is stabilized by autophosphorylation of S108 on the CBM and is dissociated when T172 on the α subunit is dephosphorylated [9]. Mutation of S108 to a phosphomimetic glutamic acid (S108E) resulted in reduced glycogen binding [31] and increased AMPK activity in response to AMP and A-769662, even in the presence of a non-phosphorylatable T172A mutation [8]. Conversely, mutation of S108 to a neutral alanine (S108A) had no effect on glycogen binding [31], but reduced AMPK activity in response to AMP and A-769662 [8]. Collectively, these findings infer that glycogen binding may inhibit AMPK activity by disrupting the interaction between the CBM and the kinase domain of the α subunit [1,9,56]. The inhibitory role of the β subunit T148 autophosphorylation on AMPK-glycogen binding has also been a focus of recent research. The mutation of T148 to a phosphomimetic aspartate (T148D) on the β1 subunit inhibits AMPK-glycogen binding in cellular systems [60]. The results from subsequent experiments in isolated rat skeletal muscle suggest that T148 is constitutively phosphorylated both at rest and following electrical stimulation, therefore, preventing glycogen from associating with the AMPK β2 subunit [61]. Further research is necessary to further elucidate the role of T148 in the context of AMPK-glycogen interactions.

Recent research has provided further structural insights into the affinity of the AMPK β subunits for carbohydrates. Isolated β2 CBM has a stronger affinity for carbohydrates than the β1 CBM, binding strongly to both branched and unbranched carbohydrates, with a preference for single α-1,6 branched carbohydrates [34]. One possible explanation for this difference is that a pocket is formed in the CBM by the T101 residue, which is unique to the β2 subunit, therefore, allowing binding to branched carbohydrates [33,34]. In addition, the β1 CBM possesses a threonine at residue 134 which may form a hydrogen bond with the neighboring W133, restricting the ability of the β1 CBM to accommodate carbohydrates, while the β2 CBM possesses a valine which does not bond with W133 [34]. This difference may explain the increased affinity of the β2 subunit for branched carbohydrates even though the 134 residue does not directly contact carbohydrates [34]. These findings indicate that the glycogen structure and branching affect AMPK binding, specifically to β1 subunits, which may dictate the inhibitory effect of glycogen observed in previous studies [34,56]. While the role of AMPK β isoform glycogen binding in the contexts of glycogen structure and branching has been investigated in vitro, it remains to be determined how these characteristics alter the dynamics of AMPK-glycogen binding in vivo.

## 4. Regulation of Cellular Energy Sensing by AMPK-Glycogen Binding

Several independent lines of evidence suggest that these physical AMPK and glycogen interactions also serve mechanistic functional roles in cellular energy sensing. A number of AMPK substrates are known to be directly involved in glycogen storage and breakdown, highlighting AMPK’s role as an important regulator of glycogen metabolism. In vitro, AMPK regulates glycogen synthesis directly via the phosphorylation and inactivation of GS at site 2 [62]. In support of this finding, AMPK α2, but not α1, knockout (KO) mice display blunted phosphorylation of GS at site 2 and higher GS activity in response to stimulation by the AMPK activator 5-aminoimidazole-4-carboxamide ribonucleotide (AICAR) in skeletal muscle [63]. Paradoxically, chronic activation of AMPK also results in an accumulation of glycogen in skeletal and cardiac muscles [38]. While these divergent outcomes appear contradictory, it has been proposed that prolonged AMPK activation leads to glycogen accumulation by increasing glucose uptake and, subsequently, by increasing intracellular G6P, a known allosteric activator of GS. This hypothesis is further supported by recent independent findings using highly specific and potent pharmacological activators demonstrating that skeletal muscle AMPK activation results in increased skeletal muscle glucose uptake and glycogen synthesis in mice and non-human primates [64,65]. This accumulation of G6P overcomes the inhibition of GS by AMPK, thereby increasing GS activity [38]. Furthermore, AMPK activation also shifts fuel utilization towards FA oxidation post-exercise, allowing glucose to be utilized for glycogen resynthesis [66]. In addition to regulating GS activity, phosphorylation of GS at site 2 by AMPK causes GS to localize to the SS and interMF glycogen pools in humans [67]. These findings have been replicated in mouse models, as an R70Q mutation of the AMPK γ1 subunit results in the chronic activation of AMPK and glycogen accumulation in the skeletal muscle interMF region [68]. This has led to the suggestion that AMPK specifically senses and responds to interMF levels of glycogen [69]; however, further research is warranted to verify this hypothesis.

An increase in GP activity has also been observed to be associated with AMPK activation induced by AICAR treatment of isolated rat soleus muscles [70,71]. However, the ability to demonstrate a direct relationship between AMPK and GP activity has been limited by the identification of several AMPK-independent targets of AICAR, including phosphofructokinase, protein kinase C, and heat shock protein 90 [72]. Further research is therefore required to determine if this speculated relationship exists and elucidate the mechanism by which AMPK may regulate GP. In contrast, there is in vitro evidence that GDE binds to residues 68–123 of the AMPK β1 subunit [73]. Mutations in this region that disrupt glycogen binding (W100G and K128Q) do not affect the binding to GDE, indicating that GDE-AMPK binding is not likely mediated by glycogen [73]. AMPK’s direct positive effect on glycogen accumulation, its known interaction with glycogen-associated proteins, and its ability to promote energy production through glucose uptake and fat oxidation when glycogen levels are low all support AMPK’s role as a cellular energy sensor. Given the limited in vivo data currently available directly linking AMPK to glycogen-associated proteins, additional studies are necessary to further understand the potential direct binding partners and effects of AMPK on the glycogen-associated proteome.

It is important to consider additional factors that may impact physical and functional AMPK-glycogen interactions. In a proteomic screen utilizing purified glycogen from rat liver, AMPK was not included in the proteins detected to be associated with glycogen [74], and this has been replicated in a complementary study in adipocytes [75]. The authors suggested that this may be due to either AMPK protein below the level of detection being able to regulate glycogen or the predominance of the AMPK β1 subunit expression in the tissues studied, as this isoform has a lower affinity for glycogen compared to the β2 subunit [74,75]. In future studies interrogating AMPK and glycogen binding and functional interactions, considerations of the β subunit isoform expression and glycogen localization, as well as sample preparation and experimental variables that may limit the preservation and detection of AMPK-glycogen binding, are warranted in future studies to build upon this strong foundation of molecular and cellular evidence.

## 5. Linking AMPK and Glycogen to Exercise Metabolism in Physiological Settings

### 5.1. Regulation of Glycogen Storage by AMPK

In the fifteen years following the discovery of glycogen binding to the CBM on the β subunit, several studies utilizing AMPK isoform knockout (KO) mouse models have provided whole-body physiological evidence of AMPK’s interactive functional roles with glycogen. Collectively, studies using AMPK α and β subunit KO mouse models have found that the ablation of AMPK alters liver and skeletal muscle glycogen content, supporting the role of AMPK in the regulation of tissue glycogen dynamics in vivo. Specifically, whole-body β2 KO mice have reduced basal glycogen levels in both liver and skeletal muscles associated with reduced muscle AMPK activity and attenuated maximal and submaximal running capacity compared to wild-type (WT) mice [76]. β2 KO mice also display reduced expression and activity of α1 and α2 subunits as well as compensatory upregulation of the β1 subunit in skeletal muscle [76]. Additional experiments utilizing this β2 KO model have demonstrated its negative impact on the whole-body and tissue metabolism and exercise capacity associated with attenuated AICAR-induced AMPK phosphorylation and glucose uptake in skeletal muscle [77]. As a result of these changes in the AMPK subunit expression and activity, it is difficult to elucidate the precise role of AMPK β2 in glycogen dynamics in this model. Similarly, muscle-specific AMPK β1/β2 KO mice display essentially no T172 phosphorylation in extensor digitorum longus (EDL) and the soleus muscle in response to electrical-stimulated contraction and have vastly reduced exercise capacity, carbohydrate utilization, and glucose uptake during treadmill running [55]. These defects were associated with reduced mitochondrial mRNA expression and reduced mitochondrial protein content [55]. Taken together, these findings suggest an important role of the β subunit in regulating AMPK activity and signaling, cellular glucose uptake and glycogen storage, mitochondrial function, and whole-body exercise capacity and metabolism. 

In addition to mouse models targeting the AMPK β subunit(s), recent studies utilizing tissue-specific α1/α2 KO mice have provided support for the functional interactive roles of AMPK and glycogen. Liver-specific AMPK α1/α2 KO mice have an impaired ability to maintain euglycemia during exercise as a result of decreased hepatic glucose output due to decreased glycogenolysis [54]. Specifically, hepatic glycogen content was reduced in KO mice following both fasting and exercise. Phosphorylation of GS was unaffected in KO mice, but a decrease in UDP-glucose pyrophosphorylase 2 content was observed, suggesting reduced glycogen synthesis due to decreased glycogen precursors rather than an altered ability to synthesize glycogen [54]. In addition, when challenged with a long-term fast, these mice had reduced hepatic glycogenolysis and were unable to maintain liver ATP concentration without AMPK activity, providing further support of AMPK’s role as an energy sensor [78]. Inducible muscle-specific α1/α2 KO mice have ablated skeletal muscle glycogen resynthesis and FA oxidation following exercise, even though glucose uptake was not affected, suggesting that AMPK functions as a switch to promote fat oxidation in order to preserve glucose for glycogen synthesis [79]. These findings indicate that AMPK can influence glycogen dynamics in physiological settings and that the ablation of AMPK activity reduces hepatic glucose output and is critical for skeletal muscle glycogen supercompensation following exercise. Collectively, studies using genetic models and pharmacological activators to date indicate that AMPK activation regulates glycogen synthesis in striated muscle (i.e., skeletal and cardiac muscles) secondary to increased glucose uptake and G6P accumulation, but not in the liver. Despite these important findings from AMPK transgenic mouse models, the precise role(s) of glycogen binding to the β subunits in the functional regulation of these physiological processes, as opposed to the ablation of the entire α subunits or β subunit(s) containing the CBM, remains to be elucidated. 

### 5.2. Roles for Glycogen Availability in the Regulation of AMPK Activity

**Figure 2:**
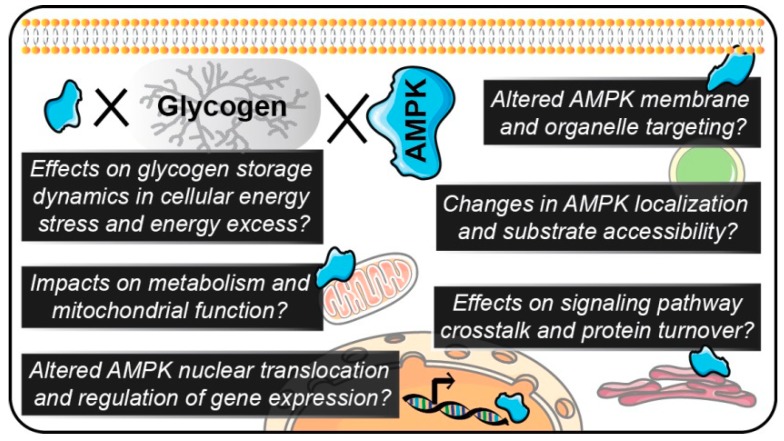
There are several potential alterations in cellular metabolism and signaling as a consequence of dysregulated AMPK-glycogen physical and functional interactions that represent key knowledge gaps in our current understanding and warrant further investigation in future studies. These potential alterations include changes in AMPK localization, translocation, substrates, and signaling pathway crosstalk, and subsequently, alterations in gene expression, cellular metabolism and glycogen storage.

A series of physiological studies have demonstrated that low glycogen availability can amplify the AMPK signaling responses and adaptations to exercise. This was originally described in rat skeletal muscles in which AICAR treatment resulted in increased AMPK α2 activity and a markedly reduced glycogen synthase activity in a glycogen-depleted state compared to a glycogen-loaded state [80]. This observation was independent of adenine nucleotide concentrations and has subsequently been replicated in human skeletal muscle following exercise [81]. Additional studies of skeletal muscles have shown reductions in AMPK α1 and α2 association with glycogen, along with increased AMPK α2 activity and translocation to the nucleus following exercise in a glycogen-depleted state [82,83]. Furthermore, the consumption of a high-fat, low-carbohydrate diet followed by one day of a high-carbohydrate diet increases the resting skeletal muscle AMPK α activity in human skeletal muscle compared to a high-carbohydrate diet alone [84], supporting glycogen’s inhibitory role on AMPK described in cell-free assays [56]. In a follow-up study, AMPK T172 phosphorylation was increased by exercise to a greater extent in the glycogen-depleted muscle than the normal glycogen repleted state [85]. Similarly, exercise in an overnight carbohydrate-fasted state resulted in increased AMPK T172 phosphorylation and the upregulation of signaling pathways involved in FA oxidation [86], while low glycogen stimulated peroxisome proliferator-activated receptor δ, a transcription factor that regulates fat utilization, in rat skeletal muscle following treadmill running [87]. Reduced glycogen availability is also associated with increases in the regulators of mitochondrial biogenesis, such as p53 and PGC-1α [88,89]. While none of these in vivo studies have directly assessed the functional role of AMPK-glycogen physical interaction, together they provide important physiological insights into how AMPK activity, subcellular localization, and signaling may be regulated by glycogen binding (Figure 2).

### 5.3. Metabolic and Glycogen Storage Diseases as Models to Investigate AMPK-Glycogen Binding 

Metabolic diseases such as insulin resistance and T2D are associated with impairments in AMPK activity, signaling, and glycogen storage dynamics. Obese patients with T2D have reduced skeletal muscle AMPK, ACC, and TBC1D4 phosphorylation following an acute bout of exercise [90]. In support of these findings, insulin resistance has been associated with suppressed AMPK activity in humans and mice [90,91], although results have been equivocal [92]. The liver-specific AMPK α1/α2 KO mice display an inability to maintain hepatic glucose output during exercise, highlighting the role of AMPK in maintaining euglycemia [54]. Skeletal muscle GS activity has also been demonstrated to be affected by insulin resistance and T2D, as there is increased phosphorylation of GS at site 2, the site phosphorylated by AMPK, which is not seen in healthy controls, resulting in nearly complete GS inactivation and dysregulation of glycogen synthesis [93]. Continued research in metabolic disease populations and rodent models can provide more insight into the significance of dysregulated AMPK and glycogen dynamics.

In addition, glycogen storage diseases provide pathophysiological models that can help provide additional insights into the influence of glycogen dynamics on AMPK. McArdle’s disease is characterized by the accumulation of skeletal muscle glycogen due to a deficiency of GP. Individuals with McArdle’s disease display higher muscle glycogen both at rest and following exercise compared to healthy controls, and an increased AMPK α2 activity and reduced GS activity in response to exercise [94]. Patients with McArdle’s disease also demonstrate increased glucose clearance and ACC phosphorylation, indicating that AMPK activity is increased in order to maintain ATP concentration by promoting glucose uptake and FA oxidation [94]. The inability to break down glycogen, when coupled with retained, albeit reduced, glycogen synthesis, likely results in glycogen accumulation and the failure to utilize this energy source during exercise in this setting of the disease. A mouse model of McArdle’s disease containing a p.R50X mutation, a nonsense mutation of nucleotide 148 in exon 1 of the GP gene, showed increased basal AMPK phosphorylation in the tibialis anterior and quadriceps muscles, associated with an increased GLUT4 content and increased AMPK-mediated glucose uptake compared to WT [95]. Following exhaustive exercise, McArdle mice display increased AMPK phosphorylation in the tibialis anterior and EDL muscles, while WT mice display no significant increase in AMPK activity [96]. While increased AMPK activity in McArdle patients and rodent models seems contrary to previous findings, the authors hypothesized that since McArdle disease results in an inability to break down glycogen, there is a subsequent increase in the AMPK activity in order to maintain an energy balance via increased glucose uptake [95,96]. Other rodent models have directly targeted muscle GS, which is affected in patients with Glycogen Storage Disease 0 [97]. Muscle-specific glycogen synthase knock-out models display increased AMPK phosphorylation [98] and markedly reduced glycogen content in skeletal muscle in the basal state, likely due to the retained capacity to break down but an inability to resynthesize glycogen [99,100].

## 6. Multidisciplinary Techniques and Models to Interrogate Roles for AMPK-Glycogen Interactions

While much remains to be discovered with regard to the molecular and cellular roles and physiological relevance of AMPK-glycogen binding, recent multidisciplinary technical research advances can be used to help address remaining knowledge gaps. For example, global mass spectrometry-based phosphoproteomics have recently revealed a repertoire of new AMPK substrates, providing additional evidence regarding the complexity and interconnection of the AMPK signaling network. A recent phosphoproteomic analysis mapping the human skeletal muscle exercise signaling network before and immediately following a single bout of intense aerobic exercise, in combination with phosphoproteomic analysis of AICAR-stimulated signaling in rat L6 myotubes, identified several novel AMPK substrates [101]. Other recent efforts have predicted and identified novel AMPK substrate phosphorylation sites via chemical genetic screening combined with peptide capture in whole cells [102], as well as affinity proteomics approach to analyzing hepatocyte proteins containing the substrate recognition motif targeted by AMPK phosphorylation [103]. Together, these complementary large-scale approaches have expanded the range of biological functions known to be regulated by AMPK. While additional substrates residing in different subcellular locations and organelles are continuing to be uncovered, the mechanisms underlying AMPK subcellular localization and targeting to substrates residing in these different organelles remains unknown. Future global, unbiased studies such as phosphoproteomics can help identify novel glycogen-associated AMPK substrates, post-translational regulation of glycogen regulatory machinery, AMPK subunit-specific regulation, and subcellular substrate targeting. Furthermore, omics-based approaches will reveal how AMPK-glycogen binding may impact other levels of biological regulation, such as the transcriptome, proteome, metabolome, and lipidome, in the contexts of exercise, metabolism, and beyond [104].

Novel AMPK fluorescence resonance energy transfer (FRET)-based sensors have recently revealed heterogeneous activity and tissue-specific roles for AMPK. These AMPK FRET sensors have permitted the spatiotemporal and dynamic assessment of AMPK activity in single cells [105], 3D cell cultures [106], and transgenic mice [107]. These biosensors build upon traditional methods to interrogate AMPK activity such as kinase assays and immunoblotting, which are limited to targeted measures of mean cellular protein phosphorylation and do not allow the spatiotemporal and dynamic assessment of AMPK activity. Electron microscopy-based approaches have also been used to visualize AMPK-glycogen association in fixed rat liver samples [58]. While improved microscopy technologies and sensors have been used to assess AMPK or glycogen localization, few studies have directly assessed AMPK-glycogen interactions. Utilizing these recent technical advancements will allow for the interrogation of AMPK-glycogen interactions and dynamics across species and physiologically relevant settings (Figure 2).

Despite the large body of research using in vitro models and physiological evidence indicating the potential functional roles for AMPK-glycogen binding, to date, there are no models that have been developed to disrupt and/or examine this physical binding directly in vivo. AMPK subunit KO models, while providing important insights into the functions of AMPK, are limited by the potential compensatory upregulation of other subunit isoforms or the disrupted stability of the AMPK heterotrimer complex (e.g., Reference [76]). In addition, directly assessing the function of β subunit glycogen binding is challenging when additional functions are altered in the presence of subunit deletion, as AMPK activity is impaired when the scaffolding β subunit is removed. The design of novel in vivo models in the future will be informed by previous molecular and cellular findings to allow direct interrogation of the functional relevance of the β subunits and CBM. Generation of novel animal models to specifically target physical AMPK-glycogen binding will provide important advances regarding its physiological significance and capability to be therapeutically targeted in vivo to modulate metabolism and the health benefits of exercise.

Finally, previous studies have primarily utilized centrifugation-based assays to detect and quantify physical AMPK-glycogen association. Novel biotechnological platforms and proximity assays will aid this investigation of AMPK-glycogen binding and AMPK’s proximity to glycogen with improved sensitivity and specificity across molecular, cellular, and physiological models. Furthermore, newly developed kinase activity reporters [108] and other non-radioactive activity assays [109] will help provide new measures of intracellular AMPK activity dynamics and complement traditional surrogate measures such as immunoblot analyses of AMPK and ACC phosphorylation. Together these technological advances expand the repertoire of available tools to monitor the range of biological processes regulated by AMPK and further our understanding of the mechanisms and physiological significance underlying AMPK-glycogen interactions.

## 7. Potential Therapeutic Relevance of Targeting AMPK-Glycogen Binding

Consistent with the therapeutic relevance of the CBM, several lines of evidence demonstrate that the CBM may play a direct functional role in AMPK conformation and activation. The CBM contains the critical S108 autophosphorylation site required for drug-induced AMPK activation in the absence of AMP [8]. Although located on opposite sides of the AMPK heterotrimer, the CBM is conformationally connected to the regulatory AMPK γ subunit and its stabilization is affected by adenine nucleotide binding (e.g., AMP) to the CBS motifs [26]. Despite physical AMPK-glycogen interaction being mediated by the β subunit, mutations in the γ subunit also result in alterations in AMPK activation and glycogen metabolism. The γ2 subunit is known to contain mutations that cause constitutive AMPK activation, resulting in glycogen storage diseases in humans. These mutations result in glycogen accumulation with coexisting deleterious effects on cardiac electrical properties that are characteristic of familial hypertrophic cardiomyopathy and Wolff-Parkinson-White syndrome [110]. In addition, gain of function mutations in the AMPK γ3 subunit predominantly expressed in skeletal muscle result in excess glycogen storage [111] as well as improvements in metabolism via increased mitochondrial biogenesis [112]. Constitutive AMPK activation associated with these γ subunit mutations promotes glycogen synthesis by increasing glucose uptake. As mentioned above, the CBM interacts with the α subunit, forming the ADaM site and stabilizing the kinase domain of the α subunit in its active formation [9,27]. However, when glycogen binds to the CBM, this interaction is destabilized, altering the ADaM site and inhibiting the AMPK activity [9]. For example, isoform-specific allosteric inhibition of AMPK has been shown to be dependent on the β2 subunit CBM in glycogen-containing pancreatic beta cells [113]. The CBM, therefore, functions as both a critical element of AMPK activation as well as a site for the allosteric inhibition by glycogen, highlighting the therapeutic potential of new drugs targeting the ADaM site.

## 8. Conclusions

AMPK is a central regulator of cellular metabolism and, therefore, possesses significant therapeutic potential for the prevention and treatment of a range of metabolic diseases. A growing body of evidence demonstrates that AMPK physically binds glycogen and this interaction can alter the conformation of AMPK, and subsequently, its activity and downstream signaling. AMPK activity subsequently regulates glycogen metabolism. Recent research has described experimental and physiological settings that impact functional AMPK and glycogen interactions, including AMPK β isoform affinity, glycogen availability, and particle size. Despite our understanding of AMPK’s relationship with glycogen, much remains to be elucidated. Further research using new technologies and experimental models can reveal additional mechanisms underlying AMPK and glycogen’s interactive roles in cellular energy sensing, exercise, and metabolism. Together, these findings will help provide insights into the physiological and therapeutic relevance of targeting AMPK and glycogen binding in health and disease.

## Figures and Tables

**Table 1:** The AMPK β subunit isoform distribution in human and mouse tissues.

Tissue	B1	B2
Human vastus lateralis	ND	~100%
Human liver	ND	~100%
Mouse extensor digitorum longus	5%	95%
Mouse soleus	18%	78%
Mouse liver	100%	ND

Adapted from References [10,28,29,30,36]. ND, nondetectable.

## Abbreviations

ACC	Acetyl-CoA carboxylase
ADP	Adenosine diphosphate
AICAR	5-aminoimidazole-4-carboxamide ribonucleotide
AMP	Adenosine monophosphate
AMPK	AMP-activated protein kinase
ATP	Adenosine triphosphate
CaMKKβ	Calcium/calmodulin-dependent protein kinase β
cAMP	Cyclic AMP
CBM	Carbohydrate-binding module
CBS	Cystathionine-β-synthase domains
EDL	Extensor digitorum longus
FA	Fatty acid
FRET	Fluorescence resonance energy transfer
G6P	Glucose-6-phosphate
GBE	Glycogen branching enzyme
GDE	Glycogen debranching enzyme
GLUT4	Glucose transporter 4
GP	Glycogen phosphorylase
GS	Glycogen synthase
interMF	Intermyofibrillar
intraMF	Intramyofibrillar
KO	Knock-out
LKB1	Liver kinase B1
mTOR	Mechanistic target of rapamycin
PGC-1α	Peroxisome proliferator-activated receptor gamma coactivator 1-α
Raptor	Regulatory associated protein of mechanistic target of rapamycin
SS	Subsarcolemmal
TBCID1	Tre-2, BUB2, CDC16, 1 domain family, member 1
TBC1D4	Tre-2, BUB2, CDC16, 1 domain family, member 4
TSC2	Tuberous sclerosis complex 2
T2D	Type 2 diabetes
UGP2	UDP-glucose pyrophosphorylase 2
WT	Wild type
